# Feasibility of Accelerometer-Based Arterial Pulse Measurement: Part I—Theoretical Analysis

**DOI:** 10.3390/s26216832

**Published:** 2026-10-29

**Authors:** Mamun Hasan, Zhili Hao

**Affiliations:** Department of Mechanical and Aerospace Engineering, Old Dominion University, Norfolk, VA 23529, USA; mhasa004@odu.edu

**Keywords:** arterial pulse measurement, single-degree-of-freedom (SDOF) system, accelerometers, baseline drift, motion artifacts, time-varying system parameters (TVSPs), instantaneous parameters

## Abstract

Despite their commercial availability and portability, accelerometers (ACCs) are much less frequently used for arterial pulse waveform (APW) measurement, and the resulting acceleration pulse waveform (${APW}_{a}$) has been much less studied than the APW measured by a tactile sensor. This paper, the first of two parts, presents a theoretical analysis of the feasibility of ACC-based arterial pulse measurement at rest, focusing on the measurement-related technical issues underlying the differences from tactile-based measurement. The tissue–contact–sensor (TCS) stack between the artery and ACC is modeled as a single-degree-of-freedom (SDOF) system to collectively capture the dynamic behavior of all the factors involved during pulse measurement, where motion artifact (MA) is quantified as low-frequency (<0.7 Hz) mechanical motion of the ACC and alters tissue prestress, resulting in time-varying system parameters (TVSPs) in the SDOF system. Theoretical relations are established among the arterial pulse signal, MA, TCS system parameters, and measured acceleration. The analysis reveals that, even in the absence of MA, the measured acceleration differs fundamentally from the displacement-domain APW because of the dynamic response of the TCS stack and the nonstationary nature of the arterial pulse signal. In the presence of MA, the measured acceleration is affected through two mechanisms: MA itself, represented as baseline drift (BD), and TVSP-induced distortion. While MA itself can be removed using conventional filtering, TVSP-induced distortion is embedded within individual arterial pulse harmonics. Numerical simulations illustrate these effects by comparing displacement- and acceleration-domain signals with and without MA and further demonstrate the distinct effects of TVSP-induced distortion on the instantaneous parameters of a harmonic. These findings provide a theoretical basis for the experimental investigation and evaluation in Part II and a mechanistic basis for MA and sensor-noise removal in ACC-measured pulse signals.

## 1. Introduction

Arterial pulse signals contain rich physiological information about the cardiovascular (CV) system, including cardiac activity, vascular properties, and cardiorespiratory coupling [1,2,3,4,5,6,7]. Consequently, noninvasive arterial pulse measurement using a variety of sensors has been extensively investigated as a low-cost, easy-to-use alternative to conventional medical instruments such as arterial tonometers and ultrasound systems [2,3,8,9]. Generally speaking, sensors for arterial pulse measurement can be classified into three categories: photoplethysmography (PPG) sensors, tactile sensors, and accelerometers (ACCs) [8,9,10,11,12,13]. Among these sensing modalities, PPG sensors were adopted for arterial pulse measurement much earlier than tactile sensors and ACCs because of their compact size, low cost, and ease of integration [2,3,14,15,16,17,18]. Their measured pulse signals are commonly referred to as PPG signals. However, owing to the optical nature of their transduction mechanism, PPG sensors are primarily used for pulse measurement at the finger, although their application to other arterial sites has attracted increasing attention in recent years [2,3,15,19]. Over the past two decades, advances in micro/nano-fabrication technologies have enabled the development of a wide variety of tactile sensors [8,10,20,21]. Because of their sensing mechanism and form factor, these sensors are mainly employed for pulse measurement at superficial arteries, particularly the radial artery (RA) and the carotid artery (CA) [10,22].

Extensive studies have been reported on arterial pulse measurement using both PPG and tactile sensors [2,8,10,18,21,22]. A tactile sensor directly measures the radial displacement of the arterial wall, from which the arterial pulse waveform (APW) can be obtained. Although a PPG sensor measures changes in blood volume rather than arterial wall motion directly, it is commonly assumed that the PPG signal is proportional to arterial diameter change [2,18,23]. Under this assumption, the PPG signal can be regarded as a surrogate measure of arterial wall radial displacement and used to derive the corresponding APW. Based on these two sensing modalities, numerous signal-processing techniques have been developed to mitigate motion artifacts (MA) and sensor noise in a measured pulse signal for extraction of the APW [2,12,13,24,25,26,27,28]. Morphological features derived from the APW have subsequently been used as arterial indices for CV assessment [29,30,31,32,33,34,35]. Effective removal of MA and sensor noise is critical for preserving the physiological information conveyed by measured pulse signals and the accuracy of the derived arterial indices [12,13,24,25,26,27,28,36].

In contrast to PPG sensors and tactile sensors, an ACC measures the radial acceleration associated with arterial wall motion rather than the APW (i.e., displacement) directly [11,12,37,38]. In principle, the radial displacement of the arterial wall, and thus the corresponding APW, can be obtained by double integration of the measured acceleration signal [37,38]. However, this approach is highly susceptible to distortion because both MA and sensor noise are accumulated during the integration process [12,39]. Consequently, ACC-based pulse measurement is typically analyzed directly in the acceleration domain to extract the APW in acceleration, ${APW}_{a}$, with its morphological features as arterial indices without performing double integration [40,41,42]. It is worth noting that the second-order time derivative (i.e., acceleration) of a PPG signal is commonly analyzed for its morphological features as arterial indices [2,30,31,32]. As such, both the second-order derivative of the PPG signal and the ACC signal represent the radial acceleration of arterial wall motion [6,12,30,43]. Consequently, they exhibit similar morphological features and can provide comparable CV information.

To the best of the authors’ knowledge, only two studies [37,44] to date have investigated ACC-based arterial pulse measurement at the CA and used double integration of the measured signals to reconstruct the APW and examine its clinical value; one additional experimental study [9] reported differences in the extracted APW between ACC-based and tactile-based measurements but did not provide an in-depth theoretical explanation of the observed differences. Beyond arterial pulse measurement, ACCs have been more widely investigated in CV assessment through chest-based measurements [45,46,47]. The resulting acceleration signal, generated by cardiac-induced mechanical vibrations of the chest wall, is commonly referred to as a seismocardiography (SCG) signal. In SCG-based assessments, electrocardiography (ECG) is often acquired simultaneously as a temporal reference because cardiac cycle timing cannot be determined from the SCG signal itself due to variations in waveform morphology [45,47,48].

ACCs have also been extensively explored in wearable CV monitoring, particularly for heart rate (HR) estimation [47,49,50]. However, wearable HR monitoring and CA-based arterial pulse measurement or chest-based SCG measurement represent fundamentally different applications. The former primarily aims to achieve robust HR estimation during daily activities, whereas the latter typically requires at-rest measurements to capture subtle morphological features of the displacement-domain pulse waveform, APW, or its acceleration-domain representation, ${APW}_{a}$. In this work, we focus on ACC-based arterial pulse measurement at the CA under at-rest conditions, with the goal of assessing the physiological information conveyed by ACC-measured signals and, thus, the feasibility of ACC-based arterial pulse measurement for CV assessment.

Recently, a single-degree-of-freedom (SDOF) system [12,13] was developed to characterize the collective effects of all measurement-related factors, including MA, on pulse signals acquired using an ACC, a PPG sensor, and a tactile sensor in both the time and frequency domains [12,13,51]. For simplicity, the ACC output in Ref. [12] was represented as a displacement signal rather than being directly analyzed in the acceleration domain. Based on this SDOF model, the influence of MA on measured pulse signals was identified, and an SDOF-based time–frequency (SDOF-TF) method [51] was developed to remove MA and sensor noise from displacement-based pulse signals using tactile sensors and PPG sensors, enabling reconstruction of the APW and derivation of time-based indices from instantaneous parameters of individual arterial pulse harmonics, including HR, heart rate variability (HRV), respiration rate (RR), and respiration modulation (RM) [51]. The effectiveness of the SDOF-TF method was validated through applications to a PPG dataset collected from subjects with and without atrial fibrillation (AF) [52] and tactile sensor measurements acquired from one healthy subject under different physiological conditions [51] and a cohort of patients with distinct CV diseases [53,54].

More recently, to investigate the marked difference in IF-derived HRV between radial artery (RA) and carotid artery (CA) measurements observed in the above tactile-based studies, the influence of MA on IF-derived HRV was investigated theoretically and experimentally [55]. The analysis revealed that large MA can substantially affect IF and, consequently, produce a non-negligible effect on the derived HRV. Motivated by this finding, the present work extends the SDOF model, the SDOF-TF method, and the associated derivation of arterial indices to investigate the feasibility of ACC-based pulse measurement for CV assessment. As Part I of this work, this paper presents a theoretical analysis of the feasibility of ACC-based arterial pulse measurement, focusing on the fundamental challenges associated with acceleration sensing and the effects of MA on ACC-measured pulse signals. The analysis demonstrates that, compared with tactile sensor measurements, ACC-based measurements face unique challenges arising from the nature of acceleration sensing and the influence of MA. In Part II of this work, an experimental study is conducted to evaluate the theoretical findings and examine their implications for the utility of ACC-based pulse measurements in CV assessment.

## 2. Materials and Methods

### 2.1. Assumptions

During arterial pulse measurement, a tissue–contact–sensor (TCS) stack is established between the ACC and the underlying artery, allowing arterial pulsation to be transmitted to the ACC. The dynamic characteristics of the TCS stack collectively represent the effects of measurement-related factors on the measured pulse signal. For simplicity, the signal transduction process within the ACC is omitted. The following assumptions are made to theoretically characterize the effects of MA on ACC-based arterial pulse measurements:

(1) Linear SDOF approximation: The TCS stack is assumed to operate within its linear regime and is represented by an SDOF system to characterize its collective dynamic behavior and the effects of measurement-related factors on the measured arterial pulse. This is an equivalent reduced-order representation of local TCS dynamics, not a literal description of the entire tissue–contact–sensor chain. Anatomical variations across measurement sites and individuals, as well as variations in sensor alignment and contact pressure, may alter the effective system parameters but do not inherently invalidate the approximation, provided that a linear SDOF system adequately represents the local dynamics under the measurement conditions.
(2) Small MA and MA-induced perturbations: The amplitude of MA is assumed to be small relative to that of the measured arterial pulse signal. MA-induced perturbations in the dynamic behavior of the TCS stack are also assumed to be small. Accordingly, the MA-induced variations in the effective SDOF system parameters, referred to as time-varying system parameters (TVSPs), are assumed to be small relative to their respective constant components. These assumptions allow the TCS stack under MA to be modeled as a time-varying SDOF system for theoretical analysis of the effects of MA on the measured arterial pulse signal.
(3) Negligible measurement-induced alteration of arterial pulsation: The mechanical interaction between the TCS stack and the underlying artery generates interaction forces that may alter arterial pulsation. For simplicity, this alteration is assumed to be negligible. Consequently, the radial displacement of the arterial wall is used as the input pulse signal acting on the TCS stack.

Based on these assumptions, the TCS stack is modeled as an SDOF system to characterize the dynamic relationship between arterial wall displacement and the measured response at the ACC. The theoretical analysis first establishes the SDOF model without MA and then extends the model to account for MA-induced variations in the system parameters. This formulation provides the mechanical basis for analyzing the effects of MA on ACC-based arterial pulse measurements.

### 2.2. SDOF Model of the TCS Stack Without MA

**Figure 1:**
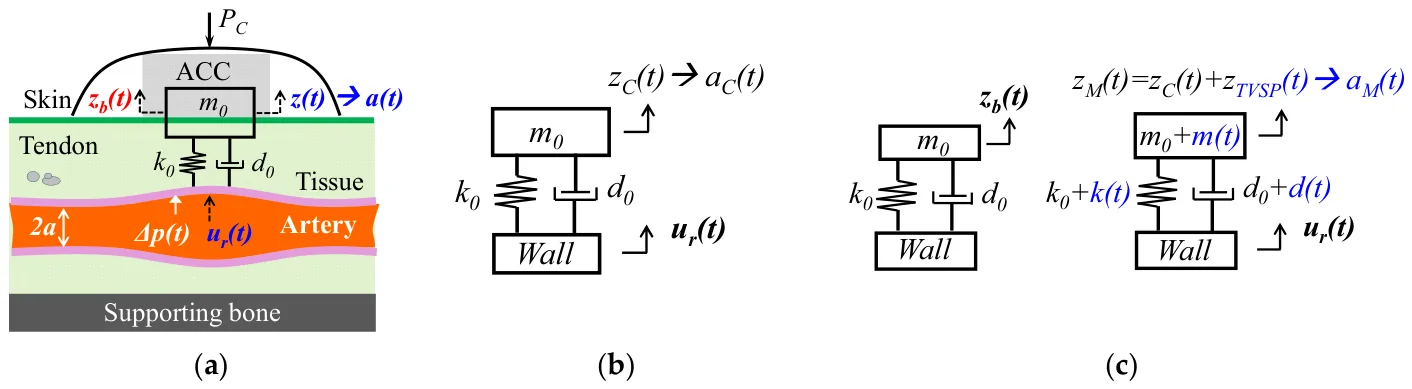
The tissue–contact–sensor (TCS) stack involved in ACC-based pulse measurement: (**a**) schematic; (**b**) an SDOF model with no MA and arterial wall radial displacement *u_r_* (*t*) as the base excitation; and (**c**) an SDOF model with MA *z_b_* (*t*) acting on the mass (or the ACC) and arterial wall radial displacement *u_r_* (*t*) as the base excitation.

As shown in Figure 1a, during pulse measurement, attaching the ACC over an artery establishes a tissue–contact–sensor (TCS) stack as the transmission path through which arterial wall radial displacement $u_{r}\left(t\right)$ is transmitted to the ACC as displacement $z\left(t\right)$ and subsequently recorded by the ACC as an acceleration signal $a\left(t\right)$. For simplicity, the transduction process is omitted, and the measured pulse signal is represented by the acceleration signal $a\left(t\right)$. The dynamic behavior of the TCS stack during pulse measurement is modeled as a single-degree-of-freedom (SDOF) system, whose parameters represent the collective effects of measurement-related factors, including ACC characteristics, ACC alignment with the artery, overlying tissue properties, and contact pressure $P_{C}$ exerted on the ACC by the attachment mechanism [12]. Additionally, MA introduces time-varying variations in these system parameters, which will be considered in Section 2.2. Detailed descriptions of the SDOF model for the TCS stack can be found in the literature [12].

As shown in Figure 1b, in the absence of MA, the SDOF system parameters are defined as follows:

$k_{0}$ and $d_{0}$: effective stiffness and damping contributions from the deformable tissue;
$m_{0}$: effective mass incorporating contributions from both the tissue and the ACC.

The applied contact pressure $P_{C}$ dictates the effective values of $k_{0}$, $d_{0}$, and $m_{0}$ through its influence on tissue prestress. Therefore, the effect of $P_{C}$ on the measured pulse signal is implicitly embedded in these system parameters.

Based on the SDOF model of the TCS stack, the arterial pulse signal $u_{r}\left(t\right)$ serves as the base excitation and is expressed as:

(1) $$u_{r}\left(t\right)=u_{r0}\cdot e^{j\phi_{u_{r}}(t)}\;\mathrm{with}\;f_{u_{r}}\left(t\right)=\frac{\omega_{u_{r}}(t)}{2\pi }=\frac{1}{2\pi }\frac{d\phi_{u_{r}}(t)}{dt}$$

where $u_{r0}$ and $\phi_{u_{r}}\left(t\right)$ represent the constant amplitude and instantaneous phase of a harmonic component, respectively, in the arterial pulse signal. For simplicity, the number of harmonics is omitted. Accordingly, the instantaneous angular frequency $\omega_{u_{r}}\left(t\right)$ is defined as the time derivative of $\phi_{u_{r}}\left(t\right)$. Due to heart rate variability (HRV), the instantaneous frequency (IF) $f_{u_{r}}\left(t\right)$ of the arterial pulse signal is inherently time-varying.

The displacement $z_{C}\left(t\right)$ of the mass (or the ACC) is related to the arterial pulse signal $u_{r}\left(t\right)$ through the SDOF system by:

(2) $$m_{0}\cdot \frac{d^{2}z_{C}(t)}{dt^{2}}+d_{0}\cdot \frac{dz_{C}\left(t\right)}{dt}+k_{0}\cdot z_{C}\left(t\right)=k_{0}\cdot u_{r}\left(t\right)+d_{0}\cdot \frac{du_{r}(t)}{dt}$$

Substituting Equation (1) into Equation (2) gives:

(3) $$z_{C}\left(t\right)=H_{C}\left(t\right)\cdot \;u_{r0}e^{j\left(\phi_{zC}\left(t\right)+\phi_{u_{r}}\left(t\right)\right)}\;\mathrm{with}\;H_{C}\left(t\right)\;e^{j\phi_{zC}(t)}=\frac{k_{0}+jd_{0}\omega_{u_{r}}}{-m_{0}\omega_{zC}^{2}+k_{0}+jd_{0}\omega_{zC}}$$

where

(4) $$\omega_{zC}\left(t\right)=\frac{d\left(\phi_{zC}\left(t\right)+\phi_{u_{r}}\left(t\right)\right)}{dt}=2\pi f_{zC}\left(t\right)\;\mathrm{with}\;f_{zC}=f_{z\_zC}+f_{u_{r}}\left(t\right)\;\mathrm{and}\;f_{z\_zC}\left(t\right)=\frac{1}{2\pi }\frac{\phi_{zC}(t)}{dt}$$

Here, $H_{C}\left(t\right)$ and $\phi_{zC}\left(t\right)$ represent the time-varying amplitude modification and phase shift of the arterial pulse signal introduced by the measurement system (i.e., the TCS stack), respectively. While $f_{zC}\left(t\right)$ is the IF of $z_{C}\left(t\right)$, $f_{z\_zC}$ is the extra term added to the IF of the $z_{C}\left(t\right)$, relative to $u_{r}\left(t\right)$, due to the dynamic response of the TCS stack, under the condition of non-zero HRV (i.e., time-varying $f_{u_{r}}\left(t\right)$).

The measured acceleration signal by the ACC is given by:

(5) $$a_{C}\left(t\right)=\Omega_{C}\left(t\right)e^{j\varphi_{aC}\left(t\right)}\cdot z_{C}\left(t\right)=\Omega_{C}\left(t\right)H_{C}\left(t\right)e^{j\left(\phi_{aC}\left(t\right)+\phi_{zC}\left(t\right)\right)}\cdot u_{r0}e^{j\phi_{u_{r}}(t)}$$

where

(6) $$\Omega_{C}\left(t\right)e^{j\varphi_{aC}\left(t\right)}=\frac{{\ddot{H}}_{C}\left(t\right)}{H_{C}\left(t\right)}-\omega_{zC}^{2}+j\left[2\frac{{\dot{H}}_{C}\left(t\right)}{H_{C}\left(t\right)}\omega_{zC}+{\dot{\omega }}_{zC}\right]$$

Accordingly, the IF $f_{aC}\left(t\right)$ of the measured acceleration signal $a_{C}\left(t\right)$ is:

(7) $$f_{aC}\left(t\right)=\frac{1}{2\pi }\frac{d(\phi_{aC}(t)+\phi_{zC}(t)+\phi_{u_{r}}(t))}{dt}=f_{z\_aC}\left(t\right)+f_{z\_zC}+f_{u_{r}}\left(t\right)\;\mathrm{with}\;f_{z\_aC}\left(t\right)=\frac{1}{2\pi }\frac{\phi_{aC}(t)}{dt}$$

Equation (7) indicates that, even in the absence of MA, the measured acceleration signal $a_{C}\left(t\right)$ does not preserve the IF of the arterial pulse signal $u_{r}\left(t\right)$. Instead, the measurement-system dynamics introduce additional terms, $f_{z\_zC}\left(t\right)$ and $f_{z\_aC}\left(t\right)$, to $f_{u_{r}}\left(t\right)$.

### 2.3. SDOF Model of the TCS Stack with MA

As shown in Figure 1c, MA, represented as a time-varying displacement $z_{b}\left(t\right)$, commonly referred to as baseline drift (BD), acts directly on the mass (i.e., the ACC):

(8) $$z_{b}\left(t\right)=z_{b0}e^{j{(\omega }_{b}t+\phi_{b})}$$

where $z_{b0}$, $\phi_{b}$, and $\omega_{b}$ denote the amplitude, initial phase, and angular frequency of $z_{b}\left(t\right)$, respectively. For at-rest measurements, the frequency of $z_{b}\left(t\right)$ is below 0.7 Hz. By changing the effective length of the TCS stack, $z_{b}\left(t\right)$ alters the system parameters through its influence on tissue prestress:

(9) $$m_{t}=m_{0}+m\left(t\right),d_{t}=d_{0}+d\left(t\right),k_{t}=k_{0}+k\left(t\right),\;\mathrm{with}\;m\left(t\right),k\left(t\right),d\left(t\right)\propto z_{b}\left(t\right)$$

where $m\left(t\right)$, $k\left(t\right)$, and $d\left(t\right)$ represent the time-varying system parameters (TVSPs). Under low-level MA, the following relations hold: $m\left(t\right)/m_{0}\ll 1,d\left(t\right)/d_{0}\ll 1,$ and $k\left(t\right)/k_{0}\ll 1$. Since TVSPs are induced by the low-frequency displacement $z_{b}\left(t\right)$, they are also characterized by low-frequency variations (<0.7 Hz). Likewise, the acceleration induced by $z_{b}\left(t\right)$ has the same low-frequency content (<0.7 Hz).

The arterial pulse signal $u_{r}\left(t\right)$, serving as the base excitation, induces displacement $z_{M}\left(t\right)$ at the mass in the presence of TVSPs [56,57]:

(10) $$m_{t}\cdot \frac{d^{2}z_{M}(t)}{dt^{2}}+\left({\dot{m}}_{t}+d_{t}\right)\cdot \frac{dz_{M}(t)}{dt}+k_{t}\cdot \;z_{M}\left(t\right)=k_{t}\cdot u_{r}\left(t\right)+d_{t}\cdot \frac{du_{r}(t)}{dt}$$

The displacement of the ACC is given by:

(11) $$z_{M}\left(t\right)=H_{M}\left(t\right)\;u_{r0}\;e^{j\left(\phi_{zM}\left(t\right)+\phi_{u_{r}}(t)\right)}\;\mathrm{with}\;H_{M}\left(t\right)e^{j\phi_{zM}\left(t\right)}=\frac{k_{t}+jd_{t}\omega_{u_{r}}}{-m_{t}\omega_{zM}^{2}+k_{t}+({\dot{m}}_{t}+d_{t})j\omega_{zM}}$$

where

(12) $$\omega_{zM}\left(t\right)=\frac{d(\phi_{zM}(t)+\phi_{u_{r}}(t))}{dt}=2\pi f_{zM}\left(t\right)\;\mathrm{with}\;f_{zM}=f_{z\_zM}+f_{u_{r}}\left(t\right)\;\mathrm{and}\;f_{z\_zM}\left(t\right)=\frac{1}{2\pi }\frac{d\phi_{zM}(t)}{dt}$$

Then, the TVSP-induced distortion $z_{tvsp}\left(t\right)$ in the displacement at the mass is:

(13) $$z_{tvsp}\left(t\right)=z_{M}\left(t\right)-z_{C}\left(t\right)=\left[H_{M}\left(t\right)e^{j\varphi_{zM}\left(t\right)}-H_{C}(t)\;e^{j\phi_{zC}(t)}\right]\cdot u_{r}\left(t\right)$$

The measured acceleration signal $a_{M}\left(t\right)$ by the ACC is therefore:

(14) $$a_{M}\left(t\right)=\Omega_{M}\left(t\right)e^{j\varphi_{aM}\left(t\right)}z_{M}\left(t\right)=\Omega_{M}\left(t\right)H_{M}\left(t\right)e^{j\left(\phi_{aM}(t)+\phi_{zM}(t)\right)}\cdot \;u_{r0}\;e^{j\phi_{u_{r}}(t)}$$

where

(15) $$\Omega_{M}\left(t\right)e^{j\varphi_{aM}\left(t\right)}=\frac{{\ddot{H}}_{M}\left(t\right)}{H_{M}\left(t\right)}-\omega_{zM}^{2}+j\left[2\frac{{\dot{H}}_{M}\left(t\right)}{H_{M}\left(t\right)}\omega_{zM}+{\dot{\omega }}_{zM}\right]$$

Accordingly, the IF of the measured acceleration signal $a_{M}\left(t\right)$ becomes:

(16) $$f_{aM}\left(t\right)=\frac{1}{2\pi }\frac{d(\phi_{aM}(t)+\phi_{zM}(t)+\phi_{u_{r}}(t))}{dt}=f_{z\_aM}\left(t\right)+f_{z\_zM}+f_{u_{r}}\left(t\right)\;\mathrm{with}\;f_{z\_aM}\left(t\right)=\frac{1}{2\pi }\frac{\phi_{aM}(t)}{dt}$$

Comparing Equations (5)–(7) with Equations (14)–(16) reveals the TVSP-induced distortion $a_{tvsp}\left(t\right)$ in the measured acceleration signal:

(17) $$a_{tvsp}\left(t\right)=a_{M}\left(t\right)-a_{C}\left(t\right)=\left[\Omega_{M}\left(t\right)\cdot H_{M}\left(t\right)e^{j\left(\varphi_{aM}\left(t\right)+\varphi_{zM}\left(t\right)\right)}-\Omega_{C}\left(t\right)\cdot H_{C}\left(t\right)\cdot e^{j\left(\varphi_{aM}\left(t\right)+\varphi_{zC}\left(t\right)\right)}\right]\cdot u_{r}\left(t\right)$$

The measured acceleration signal $a_{mea}\left(t\right)$ by the ACC can then be expressed as:

(18) $$a_{mea}\left(t\right)=a_{C}\left(t\right)+a_{b}\left(t\right)+a_{tvsp}\left(t\right)\;$$

Here, $a_{b}\left(t\right)$ represents the acceleration induced by $z_{b}\left(t\right)$, which remains in the low-frequency range (<0.7 Hz) for at-rest measurements. Accordingly, the MA effect embedded in the measured acceleration signal consists of two components: MA itself $a_{b}\left(t\right)$ and the TVSP-induced distortion $a_{tvsp}\left(t\right)$:

(19) $$\underset{MA\;embedded\;in\;measured\;signal}{a_{mea}(t)-a_{c}(t)}=\underset{TVSP-induced\;distortion}{a_{tvsp}(t)}+\underset{MA\;itself}{a_{b}(t)}$$

MA, quantified by the baseline drift, can be expressed as:

(20) $$MA:\;z_{b}\left(t\right)\to a_{b}\left(t\right)={\ddot{z}}_{b}\left(t\right)$$

where $a_{b}\left(t\right)$ represents MA in the acceleration domain. Because $a_{b}\left(t\right)$ is concentrated in the low-frequency range (<0.7Hz) and additive in nature, it can be removed using a finite impulse response (FIR) high-pass filter designed with a sharp transition band to minimize its effects on the fundamental HR harmonic. However, when the fundamental HR frequency is close to the filter transition band, the fundamental harmonic may still be affected, particularly at low HR. As will be demonstrated in Part II, the residual BD contribution to the fundamental harmonic after filtering can be further attenuated through the time–frequency analysis used in the SDOF-TF method.

In contrast, based on Equation (10), the TVSP-induced distortion $a_{tvsp}\left(t\right)$ is multiplicative and embedded within the individual harmonics of the arterial pulse because it originates from MA-induced variations in the TCS system parameters. Consequently, TVSPs introduce time-varying amplitude modulation and phase distortion into each arterial pulse harmonic, resulting in changes in both harmonic amplitudes and instantaneous frequencies. As a result, $a_{tvsp}\left(t\right)$ affects the physiological information extracted from the measured signal. In addition, compared with displacement-domain APW measurement, acceleration-domain sensing introduces further amplitude and phase changes through differentiation, which can also affect the IF and, consequently, HRV derived from the measured signal.

### 2.4. Numerical Simulation

To investigate the theoretical findings and illustrate the effects of MA and TVSPs on ACC-measured pulse signals, particularly the resulting changes in the instantaneous parameters of an arterial pulse harmonic, a simplified numerical model was developed based on the theoretical model presented in the preceding sections. For simplicity and clarity, only the third harmonic of the arterial pulse signal was considered. This single-harmonic simulation was intended to illustrate the effects of TVSPs on the three instantaneous parameters of individual harmonics in the measured arterial pulse signal: instantaneous amplitude (IA), instantaneous frequency (IF), and instantaneous initial phase (IIP), rather than to reproduce the entire APW. The third harmonic was selected because the TVSP-induced frequency modulation produces two sidebands around the harmonic frequency, allowing both sidebands to be illustrated in the frequency spectrum. Based on Equation (10), similar qualitative effects are expected for other harmonics, although their specific responses may depend on the harmonic frequency, TCS system parameters, and TVSPs. Only respiration-induced frequency modulation was incorporated because respiration is approximately time-harmonic and represents physiologically meaningful information embedded in the arterial pulse signal. Accordingly, the arterial pulse signal was defined as:

(21) $$y\left(t\right)=10\;\mathrm{mm}\cdot \cos\left(2\pi {3\cdot f}_{0}t+0.3\sin\left(2\pi 0.2t\right)\right)\;\mathrm{with}\;f_{0}=1.2\;\mathrm{Hz}$$

Yielding an IF *f*(*t*) = 3·*f*_0_ − 0.06cos(2*π*0.2*t*), where 0.2 Hz is the respiration rate (RR) and 0.06 is the respiration modulation (RM). The extracted *z_b_*(*t*) from ACC-based and tactile-based pulse measurements in Part II were used as MA inputs.

Because the TCS system parameters, effective stiffness, mass, and damping coefficient of the TCS stack, cannot be directly determined under actual arterial pulse measurement conditions, their values were assumed. First, $k_{0}$ was assumed to be one tenth of the corresponding arterial stiffness [12]. Given the clear physical implications of resonant frequency and damping factor, the values of $m_{0}$ and ${\;d}_{0}$ were assumed based on frequency ratio: $r_{0}=\frac{\omega_{0}}{\omega_{C}}=3$, where $\omega_{C}=2\pi f_{0}$ with $f_{0}=1.2$ Hz. Here, $\omega_{0}$ represents the resonant frequency of the TCS stack, while $\zeta_{0}$ = 1.5 represents the damping factor of the TCS stack. The resulting values, together with the amplitudes of the third harmonic and BD derived from an ACC-based pulse signal and a tactile-sensor-based pulse signal, are listed in Table 1.

Linear relationships were prescribed between *z_b_*(*t*) and the TVSP: *m*(*t*), *k*(*t*), and *d*(*t*) for numerical modeling purposes. Specifically,

(22) $$m(t)=m_{0}*\frac{z_{b}(t)}{max(z_{b}(t))-min(z_{b}(t))}*0.8$$

(23) $$(t)=-d_{0}*\frac{z_{b}(t)}{max(z_{b}(t))-min(z_{b}(t))}*0.7$$

(24) $$k(t)={\omega (t)}^{2}(m_{0}+m(t))-k_{0}\;\mathrm{with}\;\omega (t)=(r_{0}-\frac{z_{b}(t)}{max(z_{b}(t))-min(z_{b}(t))}*3)*\omega_{C}$$

The proportionality coefficients in these equations do not have units to ensure dimensional consistency between each parameter and its corresponding displacement-dependent term. The prescribed relationships were selected to ensure that the TVSPs remained fractions of their corresponding nominal system parameter values.

Because of the anatomical complexity of the tissue–contact–sensor chain, the actual relationships between *z_b_*(*t*) and the TVSPs may be nonlinear and depend on local tissue properties and contact conditions. The assumed linear relationships are not intended to represent experimentally established physical relationships; rather, they provide a controllable means of prescribing TVSP variations to examine their effects on the measured arterial pulse signal through numerical simulation. Additionally, nonlinear relations between *z_b_*(*t*) and the TVSPs are not expected to alter the qualitative effects of TVSP-induced distortion on the instantaneous parameters of individual harmonics, as long as TVSPs remain well below their corresponding nominal system parameter values.

All numerical simulations were performed in MATLAB R2025a. The built-in ODE45 solver was used for all time-domain simulations. The calculated displacement and acceleration at the mass, representing the displacement- and acceleration-domain measured pulse signals, respectively, were treated as the measured pulse signals. Subsequently, the Fast Fourier Transform (FFT) was applied to determine the frequency distributions of the measured signals, while Hilbert Vibration Decomposition (HVD) was applied to the measured pulse signals for time–frequency analysis (TFA) [51]. The HVD analysis of the measured pulse signal yielded an expression with three instantaneous parameters, in contrast to Equation (21) [51]:

(25) $$z_{M}\left(t\right)=A_{i}\left(t\right)\cdot \cos\left(2\pi \cdot \int f_{i}\left(t\right)dt+{ф}_{0i}\left(t\right)\right)$$

where $A_{i}\left(t\right)$, $f_{i}\left(t\right)$, and $\phi_{0i}\left(t\right)$ denote the instantaneous amplitude (IA), instantaneous frequency (IF), and instantaneous initial phase (IIP) of the *i*th harmonic, respectively. Note that the IIP was extracted from the IF using HVD to characterize respiratory-related modulation, from which RR and RM were estimated. Details of the numerical implementation and HVD algorithm can be found in the literature [51].

## 3. Results

### 3.1. Comparison of Displacement- and Acceleration-Domain Measurements Without MA

**Figure 2:**
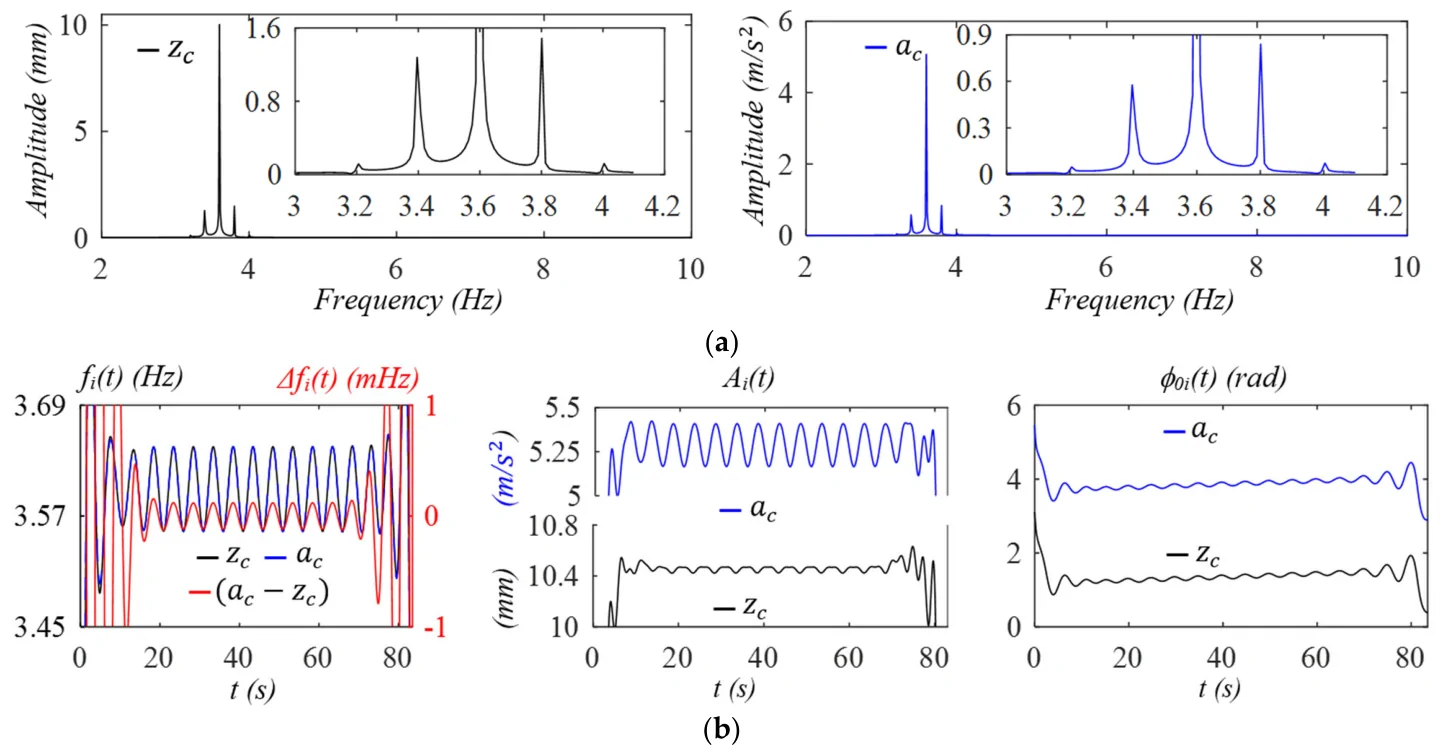
Comparison of measured *z_C_*(*t*) with *a_C_*(*t*)*:* (**a**) frequency spectra of *z_C_*(*t*) (left) and *a_C_*(*t*) (right plot); (**b**) instantaneous frequency (left), instantaneous amplitude (middle), and instantaneous initial phase of *z_C_*(*t*) and *a_C_*(*t*) (right).

Figure 2 compares *z_C_*(*t*) and *a_C_*(*t*) in terms of their frequency spectra and instantaneous parameters. The respiration term in Equation (21) produces small sidebands around the harmonic peak in both *z_C_*(*t*) and *a_C_*(*t*). Despite significant differences in amplitudes of the harmonic and its sidebands, the two signals exhibit similar overall frequency spectra. However, as shown in Figure 2b, the IFs of *z_C_*(*t*) and *a_C_*(*t*) exhibit noticeable differences. The effect of the respiration term on the IA is more pronounced in *a_C_*(*t*) than in *z_C_*(*t*). Interestingly, despite a large shift in IIP between *z_C_*(*t*) and *a_C_*(*t*), the IIP of both *z_C_*(*t*) and *a_C_*(*t*) captures the respiration term in Equation (21), and the respiration information captured in the IIP appears nearly identical in the two signals.

### 3.2. Comparison of Displacement- and Acceleration-Domain Measurements with MA

**Figure 3:**
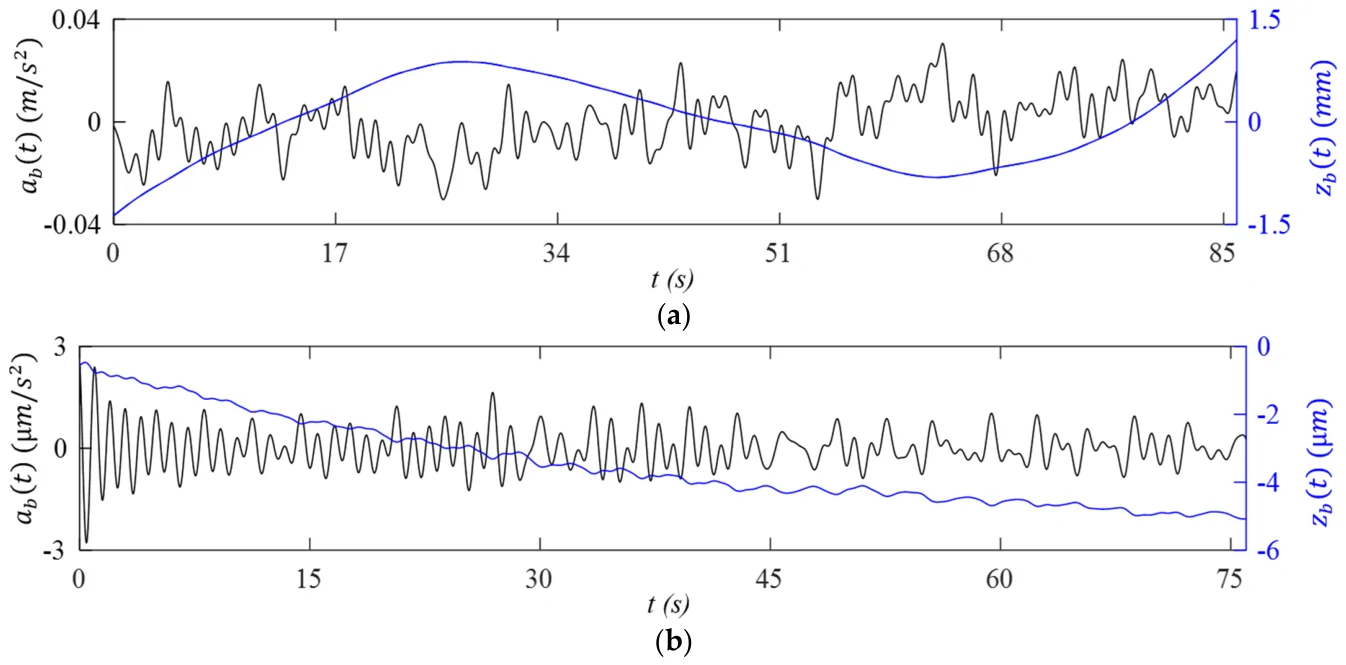
Motion artifacts (MA), quantified as BD in displacement *z_b_*(*t*) and BD in acceleration *a_b_*(*t*): (**a**) an ACC-based pulse measurement; and (**b**) a tactile-based pulse measurement.

Figure 3 compares the BD extracted from ACC-based and tactile-based measurements in Part II. Note that the BD, *a_b_*(*t*), in the ACC-based measurement was doubly integrated to obtain its displacement-domain representation, *z_b_*(*t*), while the BD, *z_b_*(*t*), in the tactile-based measurement was differentiated twice to obtain its acceleration-domain representation, *a_b_*(*t*). The *z_b_*(*t*) and *a_b_*(*t*) in the ACC-based measurement are three and four orders of magnitude larger, respectively, than their counterparts in the tactile-based measurement. Meanwhile, the *z_b_*(*t*) and *a_b_*(*t*) in the ACC-based measurement exhibit substantially larger amplitude swings but much slower temporal variations than those in the tactile-based measurement, indicating that the MA captured by the two measurement modalities differs substantially in terms of amplitude and temporal pattern.

**Figure 4:**
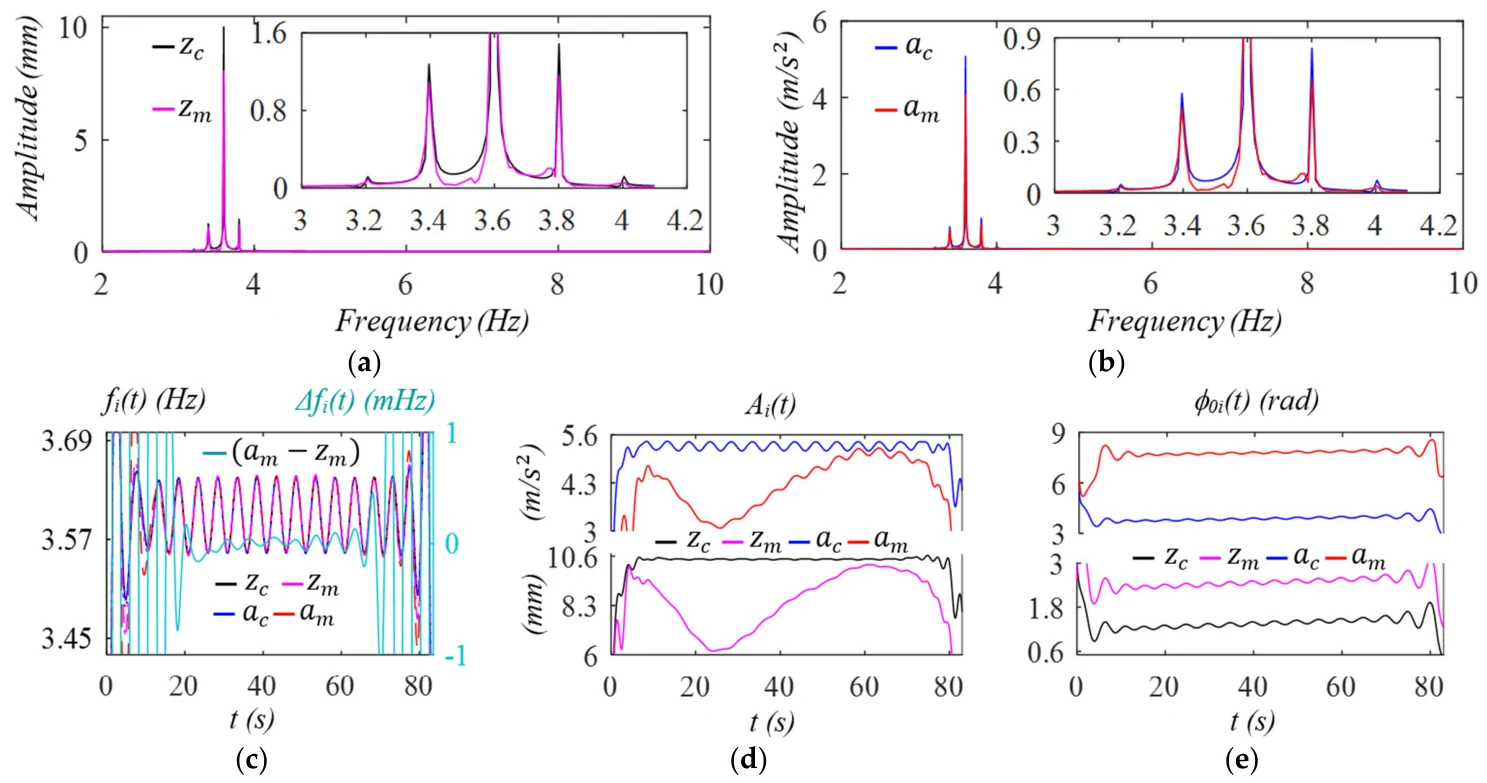
Comparison of the effects of MA in Figure 3a on measured displacement and acceleration pulse signals: (**a**) frequency spectra of $z_{C}\left(t\right)$ and $z_{M}\left(t\right)$; (**b**) frequency spectra of $a_{C}\left(t\right)$ and $a_{M}\left(t\right)$; (**c**) IF variations; (**d**) IA variations; and (**e**) IIP variations of $z_{C}\left(t\right)$, $z_{M}\left(t\right)$ $a_{C}\left(t\right)$, and $a_{M}\left(t\right)$.

Figure 4 illustrates the effect of MA on the measured pulse signals obtained from the numerical model using the BD of the ACC-based measurement shown in Figure 3a. The difference between *z_C_*(*t*) and *z_M_*(*t*) arises from *z_tvsp_*(*t*), while the difference between *a_C_*(*t*) and *a_M_*(*t*) arises from *a_tvsp_*(*t*). As shown in Figure 4a, the frequency spectra of both the measured displacement and acceleration signals are distorted by MA. Although the effects of MA on the frequency spectra appear similar for *z_M_*(*t*) and *a_M_*(*t*), their IFs exhibit noticeable differences, as shown in Figure 4b. In both *z_M_*(*t*) and *a_M_*(*t*), MA causes substantial fluctuations in the IA, with their temporal patterns following that of the BD shown in Figure 3a. Both signals capture the respiration term in Equation (20). In addition, MA causes a large shift in the IIP but has no noticeable effect on the respiration information in either *z_M_*(*t*) or *a_M_*(*t*).

**Figure 5:**
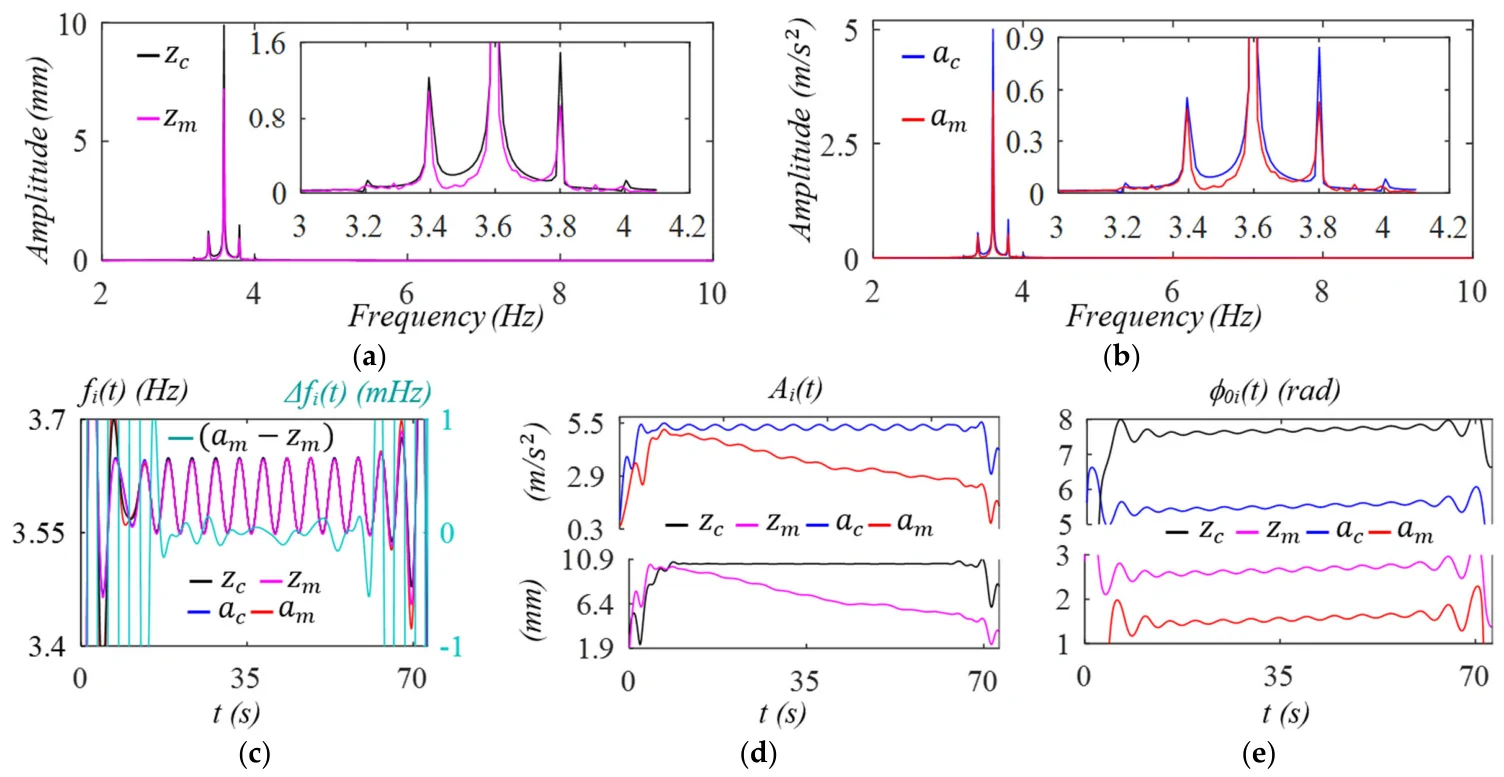
Comparison of the effects of MA in Figure 3b on measured displacement and acceleration pulse signals: (**a**) frequency spectra of $z_{C}\left(t\right)$ and $z_{M}\left(t\right)$; (**b**) frequency spectra of $a_{C}\left(t\right)$ and $a_{M}\left(t\right)$; (**c**) IF variations; (**d**) IA variations; and (**e**) IIP variations of $z_{C}\left(t\right)$, $z_{M}\left(t\right)$ $a_{C}\left(t\right)$, and $a_{M}\left(t\right)$.

Figure 5 illustrates the effect of MA on the measured pulse signals obtained from the numerical model using the BD of the tactile-based measurement shown in Figure 3b. Similarly, MA causes noticeable changes in the frequency spectrum in both *z_M_*(*t*) and *a_M_*(*t*), but the difference between their frequency spectra is not noticeable, as shown in Figure 5a. The IFs of *z_M_*(*t*) and *a_M_*(*t*), however, exhibit noticeable differences, as shown in Figure 5b. MA also causes significant fluctuations in the IA of both signals and shifts their IIP but does not affect the respiration captured in the IIP. Compared with Figure 4, the fluctuations in the IA and shifts in the IIP are much smaller in both *z_M_*(*t*) and *a_M_*(*t*) because the MA used in Figure 5 is substantially smaller than that used in Figure 4.

Table 2 shows that RR is accurately extracted from the IIP, with estimates of 12.02–12.14 breaths/min compared with the input value of 12 breaths/min, regardless of whether MA (i.e., BD) is absent or derived from ACC-based or tactile-sensor-based pulse measurements. In contrast, RM is consistently underestimated relative to the input value of 0.06. However, the difference between RM estimates derived from displacement-based and acceleration-based IIP remains negligible, regardless of whether MA is absent or derived from ACC-based or tactile-sensor-based measurements. Without MA and with ACC-based MA, RM estimates remain close to 0.054–0.055, whereas with tactile-sensor-based MA, the estimates decrease to approximately 0.044. These results suggest that RM estimation is more sensitive to MA derived from tactile-sensor-based measurements than to MA derived from ACC-based measurements, while RR estimation remains relatively robust under the simulated conditions.

## 4. Discussion

### 4.1. Fundamental Differences Between Displacement- and Acceleration-Based Pulse Measurements

Based on the theoretical analysis, even in the absence of MA, the dynamic behavior of the TCS stack and the nonstationary nature of the arterial pulse signal (i.e., non-zero HRV) result in a fundamental difference between displacement- and acceleration-based pulse measurements. The TCS stack and non-zero HRV introduce a time-varying transfer factor between the measured displacement and acceleration for individual harmonics, as shown in Equation (6). As such, the measured acceleration is no longer equal to the measured displacement divided by the squared constant angular frequency, and the HRV in the measured acceleration also differs from that in the measured displacement. The presence of MA further exacerbates the time-varying nature of the transfer factor between the measured displacement and acceleration, as shown in Equation (15).

This study is built upon our previous studies [12,51,55]. Here, we clarify the contribution of the present study relative to these previous studies. Ref. [12] was the first study to propose an SDOF model to quantify the effects of MA on measured pulse signals from accelerometers and PPG sensors in both the time and frequency domains. Importantly, the accelerometer output in Ref. [12] was represented by a displacement signal rather than directly analyzed in the acceleration domain. Ref. [51] introduced the SDOF-TF method for time–frequency analysis to remove MA and sensor noise from displacement-based pulse signals measured using tactile sensors and PPG sensors. Ref. [55] theoretically and experimentally investigated the effects of MA on HRV derived from IF in tactile sensor-based pulse measurements. Table 3 compares the differences between these studies and this study.

### 4.2. Effects of MA on the Instantaneous Parameters of the Measured Acceleration Signal

The corresponding numerical simulations validate the effects of the TCS stack and MA on the measured displacement and acceleration predicted by the theoretical analysis, regardless of the amplitude and temporal pattern of MA. The numerical results indicate that MA affects ACC-based arterial pulse measurements through two distinct mechanisms: MA itself, represented as BD, and TVSP-induced distortion. MA, represented as BD, is concentrated primarily in the low-frequency range and can therefore be removed using conventional filtering. In contrast, TVSP-induced distortion is embedded within the individual harmonics of the arterial pulse and cannot be separated from the physiological signal using conventional filtering. The significant differences in MA amplitude and temporal pattern between ACC-based and tactile-based measurements will be discussed in Part II.

Time–frequency analysis via HVD further reveals the distinct effects of MA on the instantaneous parameters of a harmonic:

(1) The IA is significantly affected by MA;
(2) The IF is only slightly affected by MA;
(3) The IIP carries respiration information related to HRV and is nearly unaffected by MA.

These findings regarding the effects of MA on the instantaneous parameters provide a mechanistic basis for the removal of MA and sensor noise from ACC-measured pulse signals in Part II.

### 4.3. Implications for Extracting Arterial Indices from Acceleration-Based Pulse Measurements

Due to its high sensitivity, an ACC must be secured to the artery using an attachment mechanism (e.g., taping). However, the contact pressure achievable with such an attachment may not be sufficiently large to ensure firm fixation of the ACC to the artery, which may also undermine the conformity to the artery during pulse measurement. In contrast, a tactile sensor is commonly secured to the artery using the fingers, allowing the contact pressure to be readily adjusted to achieve firm fixation to the artery, which also improves conformity to the artery during pulse measurement. Consequently, the TCS system parameters *k*_0_, *m*_0_, and *d*_0_ may differ between ACC-based and tactile-based measurements because of differences in the level of fixation (i.e., contact pressure). The level of fixation also affects the MA experienced during pulse measurement. As shown in Section 3, the MA amplitude and temporal pattern can differ substantially between ACC-based and tactile-based measurements.

Table 2 further shows that, although RR is accurately extracted from the IIP regardless of the MA source, RM is underestimated relative to its input value of 0.06. The difference between RM estimates derived from displacement-based and acceleration-based IIP is negligible, whereas the RM estimates are lower when MA is derived from tactile-sensor-based measurements than when it is derived from ACC-based measurements. These results suggest that the effects of MA on respiratory indices depend on the source of MA, while the extraction of RR remains relatively robust under the simulated conditions.

Differences in TCS system parameters and MA-induced distortion may contribute to differences in the measured APW and derived arterial indices, including HRV, between ACC-based and tactile-based pulse measurements. In particular, the greater MA-induced distortion in ACC-based pulse measurements may be a major factor contributing to their lower accuracy and reliability compared with tactile-based pulse measurements, which may partly explain why considerably fewer studies have been conducted on ACC-based pulse measurement than on tactile-based pulse measurement. Similarly, differences in TCS system parameters and MA among different ACCs may cause differences in their measured ${APW}_{a}$ and derived arterial indices. In Part II, we will compare the measured acceleration signals obtained using a digital ACC and an analog ACC and compare the measured pulse signals obtained using a tactile sensor and an analog ACC.

### 4.4. Validity of the Approximate Solutions to the SDOF Governing Equations

The assumptions in Section 2 establish the theoretical basis for acceleration sensing of arterial pulse signals. Equations (3) and (11) are approximate solutions to the governing equations for the TCS stack without and with MA, given in Equations (2) and (10), respectively. This section examines the residuals resulting from these approximations and establishes the conditions under which the solutions are valid. The theoretical treatment is related to previous studies on slowly varying linear systems and the responses of linear systems to modulated sinusoidal inputs [58,59,60].

Substituting Equation (3) into Equation (2) yields the residual:

(26) $$R_{C}\left(t\right)=\;u_{r0}e^{j\phi_{u_{r}}\left(t\right)}\left[m_{0}\cdot {\ddot{Q}}_{C}+\left(2jm_{0}\omega_{u_{r}}+d_{0}\right){\dot{Q}}_{C}+jm_{0}{\dot{\omega }}_{u_{r}}Q_{C}+N_{C}(\frac{D_{u_{rC}}}{D_{z_{C}}}-1)\right]\;$$

where

(27) $$Q_{C}=\frac{N_{C}}{D_{z_{C}}},D_{z_{C}}=-m_{0}\omega_{z_{C}}^{2}+k_{0}+jd_{0}\omega_{z_{C}},\;D_{u_{rC}}=-m_{0}\omega_{u_{r}}^{2}+k_{0}+jd_{0}\omega_{u_{r}},and\;N_{C}=k_{0}+jd_{0}\omega_{u_{r}}$$

When the frequency difference between the response and input is sufficiently small, such that $\omega_{z_{C}}-\omega_{u_{r}}\approx 0$, and $D_{z_{C}}$ remains away from zero, $D_{u_{rC}}/D_{z_{C}}-1\approx 0$. Consequently, the fourth term in Equation (26) is negligible. The remaining terms in the residual must be sufficiently small relative to the reference stiffness scale $k_{0}$. This yields the following sufficient conditions:

(28) $$\left|m_{0}\cdot {\ddot{Q}}_{C}\right|\ll k_{0}\;$$

(29) $$\left|\left(2jm_{0}\omega_{u_{r}}+d_{0}\right){\dot{Q}}_{C}\right|\ll k_{0}\;$$

(30) $$\;\left|jm_{0}{\dot{\omega }}_{u_{r}}Q_{C}\right|\ll k_{0}\;$$

These conditions depend on the system parameters, the arterial pulse input frequency $\omega_{u_{r}}$, and the temporal variations of $Q_{C}$ and $\omega_{u_{r}}$. Slow temporal variations of $Q_{C}$ and $\omega_{u_{r}}$ relative to the arterial-pulse oscillation can help satisfy these conditions but do not, by themselves, guarantee their satisfaction. Therefore, Equation (3) is a valid approximate solution when the conditions in Equations (28)–(30) are satisfied and the frequency-mismatch term in Equation (26) associated with $\omega_{z_{C}}-\omega_{u_{r}}\approx 0$ is negligible.

Similarly, substituting Equation (11) into Equation (10) yields the residual:

(31) $$\;\left|jm_{0}{\dot{\omega }}_{u_{r}}Q_{C}\right|\ll k_{0}\;$$

where

(32) $$Q_{M}=\frac{N_{C}}{D_{z_{M}}},D_{z_{M}}=-m_{t}\omega_{z_{M}}^{2}+k_{0}+j({\dot{m}}_{t}+d_{t})\omega_{z_{M}},\;D_{u_{rM}}=-m_{0}\omega_{u_{r}}^{2}+k_{0}+j({\dot{m}}_{t}+d_{t})\omega_{u_{r}},and\;N_{M}=k_{t}+jd_{t}\omega_{u_{r}}$$

Under low-level MA, $\omega_{z_{C}}-\omega_{u_{r}}\approx 0$ is expected. If $D_{z_{M}}$ remains away from zero, then $D_{u_{rM}}/D_{z_{M}}-1\approx 0$, making the fourth term in Equation (29) negligible. The remaining terms must be sufficiently small relative to the reference stiffness scale $k_{t}$. Accordingly, sufficient conditions for Equation (11) to be a valid approximate solution to Equation (10) are:

(33) $$\left|m_{t}\cdot {\ddot{Q}}_{M}\right|\ll k_{t}\;$$

(34) $$\;\left|\left(2jm_{t}\omega_{u_{r}}+{\dot{m}}_{t}+d_{t}\right){\dot{Q}}_{M}\right|\ll k_{t}\;$$

(35) $$\left|jm_{t}{\dot{\omega }}_{u_{r}}Q_{M}\right|\ll k_{t}\;$$

As in the case without MA, these conditions depend on the system parameters, the arterial pulse input frequency, and the temporal variations of $Q_{M}$ and $\omega_{u_{r}}$. In particular, the inertial, damping, and time-varying mass contributions in Equation (34) must be considered together; their relative magnitudes cannot be established from the parameter values alone. Although low-level MA may produce relatively slow temporal variations in the TCS system parameters, the validity of Equation (11) still requires the conditions in Equations (33)–(35) to be satisfied, together with a sufficiently small frequency-mismatch term. If these conditions are not satisfied, the approximate solution may not adequately represent the TCS response, and the residual must be evaluated to assess the approximation error.

In the numerical simulations, the responses are calculated directly from the governing equations rather than from the approximate solutions. The approximate solutions provide analytical insight into the differences between acceleration and displacement sensing. Furthermore, because the six system parameters of the TCS stack under MA—the constant parameters $k_{0}{,d}_{0},and\;m_{0}$, and the time-varying parameters $k\left(t\right),d(t)$, and m(t)—are unknown, the validity of the approximate solution cannot be established solely from the relationships among these parameters. Therefore, provided that the TCS stack operates within its linear regime, numerical simulations based on the governing equations provide a more rigorous basis for evaluating the effects of MA on acceleration and displacement sensing.

### 4.5. Study Limitations

This theoretical analysis has several limitations. First, the TCS stack is approximated as a linear SDOF dynamic system over the arterial-pulse frequency range of interest. This approximation does not imply that the entire tissue–contact–sensor chain behaves as a literal SDOF system. Rather, the SDOF model provides an equivalent reduced-order representation of the dominant mechanical input–output dynamics. Despite the anatomical complexity and potential nonlinearities at an arterial measurement site, adequate conformity between the TCS stack and the underlying artery, maintained through appropriate contact pressure, can facilitate arterial pulse transmission to the ACC. The approximation further assumes that BD (i.e., MA) varies relatively little within an individual pulse cycle compared with the arterial pulse-induced displacement. Under these conditions, the TCS stack is approximated as a linear dynamic system for theoretical analysis. Nevertheless, the effective mechanical response may depend on tissue viscoelasticity, arterial geometry, contact pressure, adhesive properties, sensor–medium coupling, sensor-axis orientation, and the frequency response of the ACC and its electronic interface. These factors may affect the adequacy of the SDOF approximation. If multiple mechanical modes contribute appreciably within the frequency range of interest, a higher-order model may be required. These considerations are consistent with the broader discussion of biomedical sensing system design in a recent review of air-coupled ultrasound technologies [61]. Accordingly, further experimental characterization is warranted to assess the adequacy of the SDOF approximation for specific anatomical measurement sites and sensor configurations.

Second, the TCS system parameters and the relationships between MA and MA-induced TVSPs are specified through modeling assumptions in the numerical simulations. Consequently, the simulated quantitative results depend on these assumptions and parameter choices, and their applicability to practical measurement conditions requires experimental validation. Nevertheless, the governing equation supports the qualitative characterization of TVSP-induced distortion in the three instantaneous parameters of individual harmonics in a measured pulse signal. Because both the nominal TCS system parameters and the three TVSPs are unknown in practical measurements, quantitative evaluation of these effects under practical measurement conditions is currently unfeasible.

Third, although respiratory information, including RR and RM, was observed to be preserved in the IIP during time–frequency analysis of simulated arterial pulse signals, the underlying physical and physiological mechanisms remain unclear. In the SDOF-TF framework, IIP is used to extract respiration-related information, whereas IF is used to calculate HR and HRV without incorporating IIP. The mathematical relationship between IF and IIP and the physical and physiological basis for their distinct roles in extracting cardiovascular and respiratory information require further investigation. However, it is worth noting that RM derived from both PPG and tactile-sensor-based arterial pulse signals has shown potential physiological significance [51,52], despite the lack of its underlying physical and physiological basis. As shown in Section 3, RR estimates derived from both ACC-based and tactile-sensor-based measurements closely match the RR of the arterial pulse signal. However, for an RM reference value of 0.06, the ACC-based and tactile-sensor-based measurements yield RM estimates of approximately 0.055 and 0.044, respectively, indicating greater underestimation with tactile-sensor-based measurements. The differences between RM estimates derived from displacement-based and acceleration-based measurements are negligible for both measurement modalities. Further theoretical and experimental studies are needed to clarify these differences and evaluate the consistency of RM estimates across measurement modalities.

Fourth, the effect of the TCS stack on the arterial pulse itself is not considered. Theoretically, the TCS stack may exert local mechanical forces on the artery, potentially altering or suppressing the underlying arterial pulse. Excluding this interaction does not invalidate the theoretical analysis of MA effects on the measured signal, provided that the arterial pulse $u_{r}\left(t\right)$ at the TCS measurement location is understood to be the pulse potentially affected by the presence of the TCS stack.

Fifth, practical factors that cannot be fully anticipated through theoretical analysis may affect ACC-based pulse measurement. These factors include sensor noise, measurement sensitivity, MA levels relative to the measured arterial pulse signal, and other sources of signal distortion. Their effects on measurement feasibility require experimental investigation, which is addressed in Part II under the conditions studied.

Overall, this theoretical analysis provides a basis for understanding the fundamental mechanical effects of MA on ACC-based arterial pulse measurements. Provided that adequate conformity between the TCS stack and the underlying artery is achieved and MA remains low relative to the arterial pulse signal, a linear SDOF model can provide a sufficient reduced-order representation of local TCS dynamics despite variations in effective system parameters across measurement sites and individuals. The validity of the modeling assumptions and the influence of practical measurement factors on the feasibility of ACC-based pulse measurement are further investigated experimentally in Part II.

## 5. Conclusions

This paper presents a theoretical analysis of ACC-based arterial pulse measurement, demonstrating fundamental differences between displacement- and acceleration-based measurements even in the absence of MA. These differences arise from the dynamic behavior of the TCS stack and the nonstationary nature of the arterial pulse signal, which together introduce a time-varying transfer factor between the two measurement domains. The analysis further demonstrates that MA affects ACC-based pulse measurements through two mechanisms: MA itself, represented as BD, and TVSP-induced distortion. While MA itself is additive and can be removed by conventional filtering, TVSP-induced distortion is embedded within individual arterial pulse harmonics. Time–frequency analysis further shows that the IA is highly sensitive to MA, whereas the IF is only slightly affected and the IIP remains nearly unaffected while preserving the respiration-related component of HRV. These findings provide a theoretical basis for the experimental investigation in Part II and a mechanistic foundation for developing MA and sensor-noise mitigation methods. Part II further evaluates the validity of the modeling assumptions and the influence of practical measurement factors on the feasibility of ACC-based arterial pulse measurement.

## Figures and Tables

**Table 1:** Constant values of the TCS system parameters and BD amplitudes used in the numerical model. The BD amplitude is defined as the maximum minus the minimum of *z_b_*(*t*) in ACC measurements, ${Amp}_{BD-ACC},$ and as the amplitude of *z_b_*(*t*) in tactile sensor measurements, ${Amp}_{BD-TAC}$.

Parameter	$m_{0}$ (kg)	$d_{0}$ (kg/s)	$k_{0}$ (N/m)	${Amp}_{3rd-Har}$ (mm)	${Amp}_{BD-ACC}$ (mm)	${Amp}_{BD-TAC}$ (mm)
Value	0.0046	0.31	2.37	10	2.5670	0.0046

**Table 2:** RR and RM values derived from the IIP in Figure 4 and Figure 5, with input values of RR = 12 breaths/min and RM = 0.06 in Equation (21).

	Without BD		ACC-Based BD		Tactile-Sensor-Based BD	
	$z_{c}$	$a_{c}$	$z_{m}$	$a_{m}$	$z_{m}$	$a_{m}$
RR (breaths/min)	12.09	12.09	12.09	12.14	12.02	12.02
RM (a.u.)	0.0545	0.0543	0.0550	0.0548	0.0444	0.0444

**Table 3:** Comparison of the present study with Refs. [12,51,55].

Reference	Primary Focus	Main Contribution	Distinction of the Present Study
Ref. [12]	MA effects in ACC- and PPG-based pulse measurements	Proposed an SDOF model to quantify MA effects: BD and TVSPs, on measured pulse signals in the time and frequency domains; the ACC output was represented as a displacement signal.	The present study directly examines TVSP effects on pulse signals measured in the acceleration domain and compares them with the effects on displacement-based pulse signals.
Ref. [51]	Time–frequency analysis of pulse signals measured using tactile sensors and PPG sensors	Introduced the SDOF-TF method for the SDOF-based time–frequency analysis to remove MA and sensor noise from displacement-based pulse signals.	The present study focuses on the theoretical effects of TVSPs on instantaneous parameters of harmonics, comparing ACC-based and displacement-based measurements.
Ref. [55]	MA effects on HRV derived from IF in tactile sensor-based pulse measurements	Investigated how MA affects IF-derived HRV in displacement-based pulse measurement.	The present study examines TVSP effects on IA, IF, and IIP, including differences between acceleration-based and displacement-based measurements.
Present study	TVSP-induced distortion in ACC-based pulse measurements	Theoretically analyzes TVSP effects within an SDOF framework and compares their effects on instantaneous parameters obtained through acceleration and displacement sensing.	Establishes a theoretical basis for understanding differences in TVSP effects between acceleration sensing and displacement sensing.

## Data Availability

The data that support the findings of this study are available upon reasonable request from the corresponding author. The data are not publicly available due to privacy or ethical restrictions.

## Abbreviations

The following abbreviations are used in this manuscript:

ACC	Accelerometer
BD	Baseline drift
SDOF	Single degree-of-freedom
SDOF-TF	Single degree-of-freedom time–frequency
TVSP	Time-varying system parameters
MA	Motion artifacts
TCS	Tissue–contact–sensor
HVD	Hilbert vibration decomposition
HRV	Heart rate variability
IF	Instantaneous frequency
IA	Instantaneous amplitude
IIP	Instantaneous initial phase
